# The Impact of School Closure on Adolescents’ Wellbeing, and Steps towards to a New Normal: The Need for an Assessment Tool Update?

**DOI:** 10.3390/adolescents1030027

**Published:** 2021-09-05

**Authors:** Leena Paakkari, Didier Jourdan, Jo Inchley, Minna Torppa

**Affiliations:** 1 Faculty of Sport and Health Sciences, University of Jyväskylä, 40014 Jyväskylä, Finland; 2 UNESCO Chair and WHO Collaborating Center in Global Health & Education, University Clermont Auvergne, 63407 Chamalières, France; 3 MRC/CSO Social and Public Health Sciences Unit, University of Glasgow, Glasgow G7 7HR, UK; 4 Department of Teacher Education, University of Jyväskylä, 40014 Jyväskylä, Finland

**Keywords:** school closure, COVID-19, wellbeing, assessment, adolescents

## Abstract

Close to 200 countries have implemented school closures to decrease the spread of the COVID-19 coronavirus. Though the closures have seemed necessary, their effects on the wellbeing of children and adolescents have raised serious concerns. To truly understand the impact of such disruption on young people’s wellbeing, and their views on how to move towards a new normal, we must adopt different approaches to gather the data to secure children’s and adolescents’ rights to be heard in the issues that concern their lives. Current ways to examine the impacts of school closure have been dominated by gathering information concerning the children and adolescents, using mainly existing wellbeing indicators and related questionnaire surveys. Although such sources of information are important, they provide limited understanding of how children and adolescents have experienced school closures, especially if they have been produced using measures developed purely by adults. There is a need for information produced by children and adolescents themselves, which may require going beyond existing and pre-COVID theoretical wellbeing frameworks. By capturing information produced by children and adolescents, we can more effectively guide the development and evaluation of public health policies and identify solutions to mitigate the negative impacts of school closure, or to acknowledge the possible positive effects, and respond accordingly.

Close to 200 countries implemented school closures in 2020–2021 as a means to decrease the spread of the COVID-19 coronavirus. Already by August 2020, the strategy had affected almost 1.6 billion school-aged children [1]. Although the closures were deemed necessary, their effects on the wellbeing of children and adolescents have raised serious concerns [2]. According to the World Health Organization [3], “schools should be among the last places to be closed”. The crisis has highlighted the need to weigh the public health benefits of closures against the negative impacts on education, health and wellbeing among adolescents. It has further revealed important gaps in current approaches to the assessment of adolescent wellbeing [4].

The assessment of a person’s wellbeing is directly dependent on the context in which he or she lives. Thus, assessment tools (e.g., questionnaires) for children and adolescents cannot be a simple “vocabulary adjustment” of those for adults. The tools must integrate what shapes the current lives of the respondents, such as school experiences, relationships with peers and adults, digital spaces, and safety. Although listening to children’s voices has been emphasized in scientific [4] and political discussions on school closure [3], current efforts to inform policy development have mainly focused on gathering wellbeing information concerning adolescents as opposed to information produced by adolescents [5]. The latter approach aims to investigate wellbeing “through children’s experiences and conceptions” whereas the former “heavily accentuates the point of view of adults” and is measured mainly by statistics on risks and problems [5]. Overall, adolescent health and wellbeing indicators have tended to focus on health deficits rather than positive health (e.g., injury and violence, and risks for non-communicable diseases [6]).

Especially during the first waves of the coronavirus, the information concerning children and adolescents with regard to wellbeing during school closures was largely obtained via population-level indicators. These included quantitative estimates of poverty, school drop-out, malnutrition and child maltreatment, and the number of infections. Such indicators are mostly based on the ways in which adults define wellbeing (including wealth, safety, and objectively measured health) and on elements recognized as appropriate to evidence-informed policy-making. Wellbeing is then assessed “through the extent to which these ‘needs’ are satisfied” [7] or through changes in the measured indicators during school closure.

Several countries have collected wellbeing data using self-administered questionnaires completed by adolescents for the purposes of national decision-making. These questionnaires exemplify methods closer to data produced by adolescents themselves, since they collect subjective evaluations of children’s own lives [7]. However, adolescents’ experiences of school closure can differ from those of adults. If questionnaires include questions and items developed purely by adults, they are likely to be biased towards adult definitions of wellbeing. The mismatch between adults’ views and adolescents’ views may be particularly pronounced in the completely novel situation brought about by school closures in the midst of a global pandemic.

Though the various indicators and questionnaires produced so far offer valuable data in monitoring the national, regional, and global situation, they give little understanding of “what it means to be a child in [a given] situation—understanding which cannot be derived from quantitative data alone” [8]. For global and regional policy-making purposes, there is an urgent need for complementary data to build a deeper understanding of the current situation and also to develop new global and regional wellbeing indicators along with other monitoring tools. Country-specific indicators are also needed to address particular issues relevant for child wellbeing within different national contexts.

The need for information produced by adolescents points towards child-centered co-produced research methods that go beyond existing and pre-COVID theoretical wellbeing frameworks. The COVID-19 pandemic has tested our understanding of wellbeing, and highlighted that no single definition, theory, or model can capture the full range of experiences of children and adolescents. The need for continued wellbeing assessment will not end when the acute pandemic situation is resolved. Young people have been affected in a wide variety of ways, and it is likely that the impact on their wellbeing will be long-lasting, particularly for those experiencing long-term or severe disruption to their education or to those living in challenging home environments. To truly understand the impact of such disruption on young people’s wellbeing, and their views on how to move towards a new normal, a wider variety of data collection methods is needed, including, for example, interviews, drawings, or photographs taken by the young people themselves.

Unicef [9], for instance, has planned valuable research to examine children’s experiences and views of COVID-19, using mainly qualitative methods. The intention is to genuinely involve children in planning responses to the current situation—and that is a welcome development. However, it appears that when the intention is to assess the impact of school closure on adolescents, it will be done via scholarly literature reviews and surveys see [9]. Without extensive coverage of the information produced by adolescents during and after the COVID-19 crisis, there is a risk that any reviews or discussions on wellbeing will fail to encompass the broad range of adolescents’ perceptions and emotions regarding the impact of school closure. This will limit understanding of the factors to be taken into account in planning post-COVID-19 schooling, when the aim will be to tackle the wellbeing issues and wellbeing disparities resulting from COVID, and further, to develop schooling on the basis of positive experiences and lessons learned. The needs of children may not be met if policy makers fail either to mitigate the negative impacts of school closure or to acknowledge the possible positive effects, and make plans accordingly. As noted recently, “it is imperative that we validate the experiences of the young during this global crisis” [2]. Furthermore, as stated by the OECD (Paris, France) [10], the wellbeing indicators should echo children’s views and perspectives by “using information on children’s own priorities and perspectives on what should be measured when it comes to their wellbeing” (p. 58). To that end, assessment tools need to be updated on the basis of scientific evidence on the determinants of genuine youth participation.

The current crisis provides a timely reminder of the importance of allowing adolescents’ voices to inform decisions on issues that affect their lives. Data obtained in traditional ways need to be complemented through innovative and engaging wellbeing assessment tools for school-level, regional, and global policy formation purposes. By capturing the views and experiences of adolescents in their own words, we can more effectively guide the development and evaluation of public health policies, and “encourage, empower, and engage them [children and adolescents] in forming creative solutions for a new normal” [11].
